# Increased risk of thyroid disease in patients with Sjogren's syndrome: a systematic review and meta-analysis

**DOI:** 10.7717/peerj.6737

**Published:** 2019-03-19

**Authors:** Xin Sun, Li Lu, Yanbin Li, Rong Yang, Ling Shan, Yang Wang

**Affiliations:** Department of Endocrinology and Metabolism, People's Hospital of Liaoning Province, Shenyang, China

**Keywords:** Thyroid disease, Sjogren's syndrome, Incidence

## Abstract

**Background:**

Sjogren’s syndrome (SS) is a chronic autoimmune epithelitis characterized by disruption of epithelial cells, ensuing lymphoplasmocytic infiltration of exocrine glands, and subsequent dryness of the mouth and eyes. Individuals with SS are more likely to have the thyroid disease. However, this association remains controversial. This meta-analysis aimed to evaluate the risk of thyroid disease in patients with SS.

**Methods:**

We performed this systematic review by searching both English and Chinese literature databases. Random- or fixed-effects models were used to summarize the association between thyroid disease and SS. The results were subjected to meta-analysis with odds ratios (ORs) and corresponding 95% confidence intervals (CIs).

**Results:**

The eight articles in this meta-analysis included 988 SS cases and 2,884 controls. Overall, the risk of thyroid disease in patients with SS was significantly increased compared with controls (OR, 3.29; 95% CI [2.08–5.21]). The risk of autoimmune thyroid disease (AITD) and non-AITD were also higher in patients with SS than in controls (OR, 3.48; 95% CI [1.59–7.63]; and OR, 2.90; 95% CI [1.51–5.57], respectively).

**Conclusions:**

To the best of our knowledge, this systematic review is the first to demonstrate that the risk of thyroid disease was increased in SS compared to controls, suggesting that SS patients should be screened for thyroid disease.

## Introduction

Sjogren’s syndrome (SS) is an autoimmune disease involving the exocrine glands. Its typical clinical manifestations are persistent dry mouth and eyes. Some female patients may even have vaginal dryness (Baldini et al., 2018). It may occur alone as primary SS (pSS) or be associated with other connective tissue diseases as secondary SS (sSS). The disease can lead to multiple system and organ damage, especially the lungs, kidneys, and blood system (Patel & Shahane, 2014). The initial study suggested that SS with thyroid disease is not common, but as the incidence of SS continues to rise, so does the incidence of concomitant thyroid disease.

The first report on the possible association between SS and thyroid disease was described in the 1960s (Jara et al., 2007). Whaley et al. (1973) were unable to show an association between autoimmune thyroid disease (AITD) and SS. Perez et al. (1995) studied the thyroid function of 33 pSS patients and found thyroid disease in 45%, AITD in 24%, hypothyroidism in 33%, and hyperthyroidism in 6%. Overall, the prevalence of AITD in SS has been assessed by several heterogeneous studies over time and apparently varies from 3% to 40% (Punzi et al., 1996; Biro et al., 2006; Zeher et al., 2009).

As mentioned above, the risk of AITD may be higher in SS patients than in controls. However, there is a lack of agreement about the prevalence of thyroid disease and non-AITD in SS patients. This study aimed to evaluate the risk of different thyroid diseases in patients with SS.

## Methods

This systematic review and meta-analysis was registered in PROSPERO (registration number: CRD42018105423). A completed preferred reporting items for systematic review and meta-analyses checklist is presented in the Supplement File.

### Search

We searched for epidemiological studies in the PubMed, Web of Science, Embase, and China National Knowledge Infrastructure (CNKI) electronic databases with the words “Sjogren’s syndrome” or “SS” in combination with the terms “thyroid disease,” “thyroiditis,” “thyroid antibody,” “hypothyroidism,” or “hyperthyroidism” in the title and abstract. Two authors (LL and YBL) performed the search together. All studies published from January 1990 to August 2018 were searched. In addition, the reference lists of the retrieved articles were examined to identify additional eligible studies. Unpublished studies were not retrieved. The search languages were limited to English and Chinese.

### Inclusion criteria

To satisfy the analysis requirements and reduce selection deviation, the included studies met the following inclusion criteria: (1) patients with SS met standard diagnostic criteria, such as European criteria or the American–European Consensus Group criteria for SS (Vitali et al., 1993, 2002); (2) a case-control or retrospective study design was used; (3) sufficient data for cases and controls were provided to enable calculation of the odds ratio (OR) with a 95% confidence interval (CI) and *P*-value. Reviews, case reports, letters, and animal research were excluded from this meta-analysis.

### Data extraction

The following information was extracted from the included studies by two independent reviewers (LL, YBL): first author, year of publication, region, diagnostic criteria, number of cases and controls, and details of thyroid disease in the cases and controls. Discussion between the two reviewers resolved any differences. If no consensus could be reached, another reviewer (XS) resolved the conflict.

### Risk of bias in included studies

Two independent reviewers, who were not blinded to the authors or journals, assessed the risk of bias in the included studies using the newcastle-ottawa scale (NOS) (Wells et al., 2014). The NOS is recommended by the Cochrane Handbook for systematic reviews of interventions. Each included study was judged in terms of three domains using the “star system”: representativeness of the study group selection (four items), comparability of the groups (two items), and ascertainment of either the exposure or outcome (three items). NOS scores range from zero to nine stars. The two reviewers evaluated the studies, compared their findings, and resolved any differences.

### Statistical analysis

We employed a systematic analysis to calculate the risk of thyroid disorder in patients with SS by the pooled OR with the corresponding 95% CI. A random- or fixed-effects model was selected to summarize the risk of thyroid disease in SS patients compared to controls. Heterogeneity among studies was assessed using Cochran’s *Q*-test and the *I*^2^ statistic, which shows the percentage of variation among studies. If the data showed low or moderate heterogeneity (*I*^2^ < 50%), a fixed-effects model was used; otherwise, a random-effects model was used. Additionally, a sensitivity analysis was performed to examine the influence of any particular study on the pooled estimate. Publication bias was evaluated using Begg’s test. The significance level was set at *P* < 0.05. All statistical analyses were performed using STATA version 12.0 (Stata Corp LP, College Station, TX, USA).

## Results

**Figure 1:**
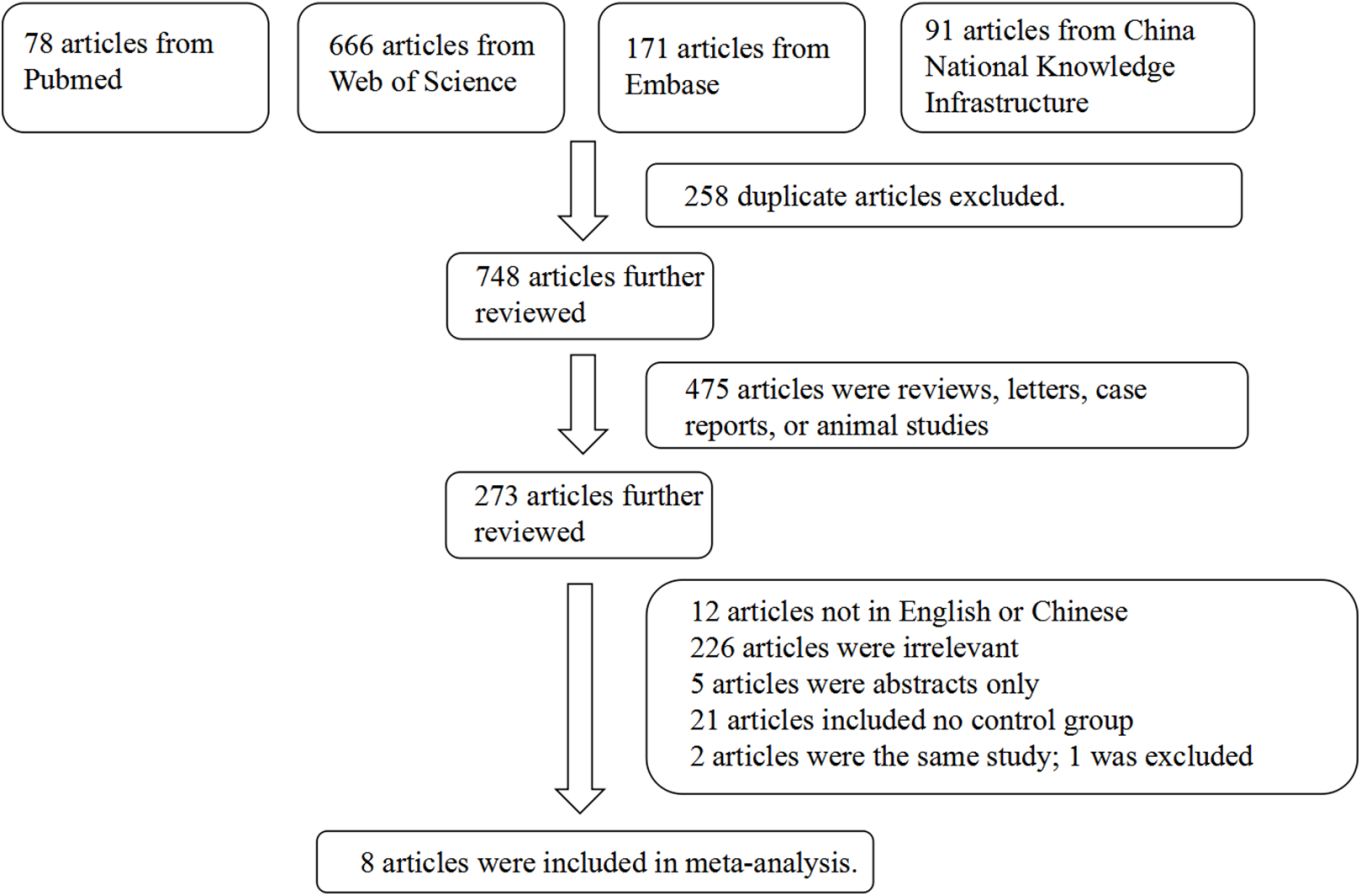
Flow chart showing the detailed procedure for study inclusion or exclusion. The initial search retrieved 1006 articles from PubMed, Web of Science, Embase, and CNKI. After selection, eight articles were included.

The initial search retrieved 78 articles from PubMed, 666 from Web of Science, 171 from Embase, and 95 from CNKI. After selection, eight articles were included. Two articles included both pSS and sSS patients, while the other six articles included only pSS patients (Ramos-Casals et al., 2000; Al-Hashimi et al., 2001; Ruggeri et al., 2002; D’Arbonneau et al., 2003; Tunc, 2004; Al-Awadhi et al., 2007; Lu et al., 2013; Shi et al., 2015). The main reasons for inclusion during the full-text screening are shown in Fig. 1. We included these eligible studies for our meta-analysis, which included 988 SS cases and 2,684 controls. The characteristics of the selected studies are summarized in Tables 1 and 2. Based on the NOS, the quality of the research contained in this meta-analysis is acceptable; therefore, we did not exclude any articles.

**Table 1:** Characteristics of the studies included in the meta-analysis.

Study	Study period	Design	Region	Criteria	Case(n)	Control(n)	Case factor	Control factor	Outcome
Ramos-Casals et al. (2000)	–	Case-control	Mexico	EC	160	75	147 F, 13 M; 23–87 years; all had pSS	66 F, 9 M; 23–80 years; similar sex, age, demographic profiles	Thyroid disease, AITD, hyperthyroidism, hypothyroidism
Al-Hashimi et al. (2001)	–	Case-control	USA	EC	169	44	24–85 years (59.9 ± 13.3 years); 92% patients had pSS	20–77 years (48.3 ± 13.3 years)	Thyroid disease
Ruggeri et al. (2002)	1998–1999	Retrospective study	Italy	EC	20	20	19 F, 1 M; 51 ± 12 years; all patients had pSS	17 F, 3 M; 38 ± 6 years; collagenoses other than SS and RA	AITD
D’Arbonneau et al. (2003)	1985–2000	Case-control	France	EC	137	120	19–73 years (53.6 ± 11.9 years); all patients were women with pSS	Patients with sciatica and OA were controls; all controls were female. No controls had RF, ANA, or anti-Ro/SSA and anti-La/SSB antibodies; they were age-matched to pSS patients	Thyroid disease, AITD, hyperthyroidism, hypothyroidism
Tunc (2004)	–	Case-control	Turkey	EC	65	53	53 patients had pSS (51 F, 2 M; mean age, 51.8 years); 12 patients had sSS (12 F, 0 M; mean age, 57.3 years)	53 healthy controls from among hospital staff and their relatives age- and sex-matched to patients with pSS	AITD
Al-Awadhi et al. (2007)	–	Case-control	Kuwait	AECG	25	577	23 F, 2 M; 45.8 ± 1.2 years; all patients had pSS	377 F, 200 M; 36.8 ± 10.3 years; apparently healthy controls aged ≥18 years were recruited from a house to house survey of Kuwaiti households No controls had any prior history or clinical diagnosis of rheumatic disease or acute systemic disease; none were on medications known to affect thyroid function	Thyroid disease, AITD, hyperthyroidism, hypothyroidism
Lu et al. (2013)	2005–2010	Retrospective study	Taiwan	AECG	343	1,735	All patients were female with pSS; 20–89 years (53.9 ± 13.7 years)	For each pSS case, five controls were randomly selected from the 2000 Longitudinal Health Insurance Database frequency-matched by 10-year age interval, sex, and year of index date	Thyroid disease, hyperthyroidism, hypothyroidism
Shi et al. (2015)	2010–2014	Case-control	China manland	AECG	69	60	All patients were female with pSS; 20–70 years	OA patients were controls without RF, ANA, or anti-Ro/SSA, and anti-La/SSB antibodies and were age- and sex-matched to the pSS patients	Thyroid disease, AITD, hyperthyroidism, hypothyroidism

**Notes:**

Criteria for the classification of Sjogren’s syndrome are those supported by the European Communityy (Vitali et al., 1993) or by the American–European Consensus Group (Vitali et al., 2002).

SS, Sjogren’s syndrome; pSS, primary Sjogren’s syndrome; AITD, autoimmune thyroid disease; OA, osteoarthrosis; RF, rheumatoid arthritis.

**Table 2:** Details of thyroid disease in patients with sjogren’s syndrome and controls in the study (n).

Study	Thyroid disease		AITD		Non-AITD		Hyperthyrodism		Hypothyrodism	
	Case	Control	Case	Control	Case	Control	Case	Control	Case	Control
Ramos-Casals et al. (2000)	58	20	32	13	26	7	12	1	31	10
Al-Hashimi et al. (2001)	56	5								
Ruggeri et al. (2002)	11	0	11	0						
D’Arbonneau et al. (2003)	48	11	39	8	9	3	1	0	22	3
Tunc (2004)	6 in pSS	4	2 in sSS	4						
Al-Awadhi et al. (2007)	8	39	3	18	5	21	0	1	5	18
Lu et al. (2013)	71	188					34	74	13	28
Shi et al. (2015)	23	6	19	4	4	2			11	1

**Note:**

AITD, autoimmune thyroid disease; pSS, primary Sjogren’s syndrome; sSS, secondary Sjogren’s syndrome.

### Results of the meta-analysis

**Figure 2:**
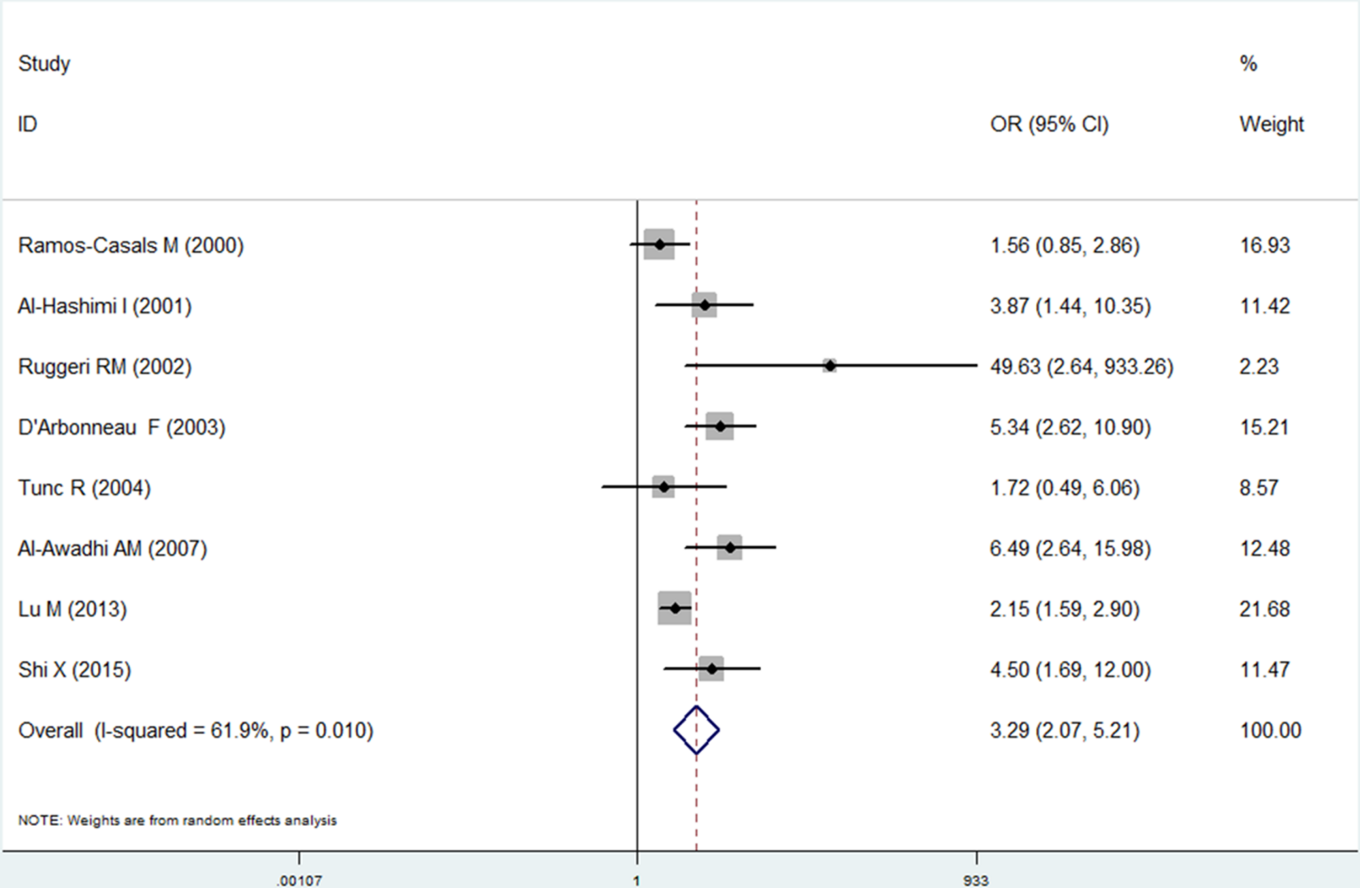
Forest plots of the frequency of thyroid disease in patients with Sjogren’s syndrome versus controls. Diamond, pooled odds ratio (OR), and 95% confidence interval (CI).

The results of the meta-analysis indicated that the risk of thyroid disease was significantly higher in patients with SS than in the controls (OR, 3.29; 95% CI [2.08–5.21]). The forest plots of the frequency of thyroid disease in patients with SS compared with controls are presented in Fig. 2. Moreover, the risk of thyroid disease was also significantly higher in patients with pSS than in controls (OR, 3.24; 95% CI [1.93–5.44]). In addition, we noticed moderate heterogeneity (*I*^2^ = 61.9%, *P* = 0.01) in the meta-analysis. We performed further analysis for different thyroid diseases.

**Figure 3:**
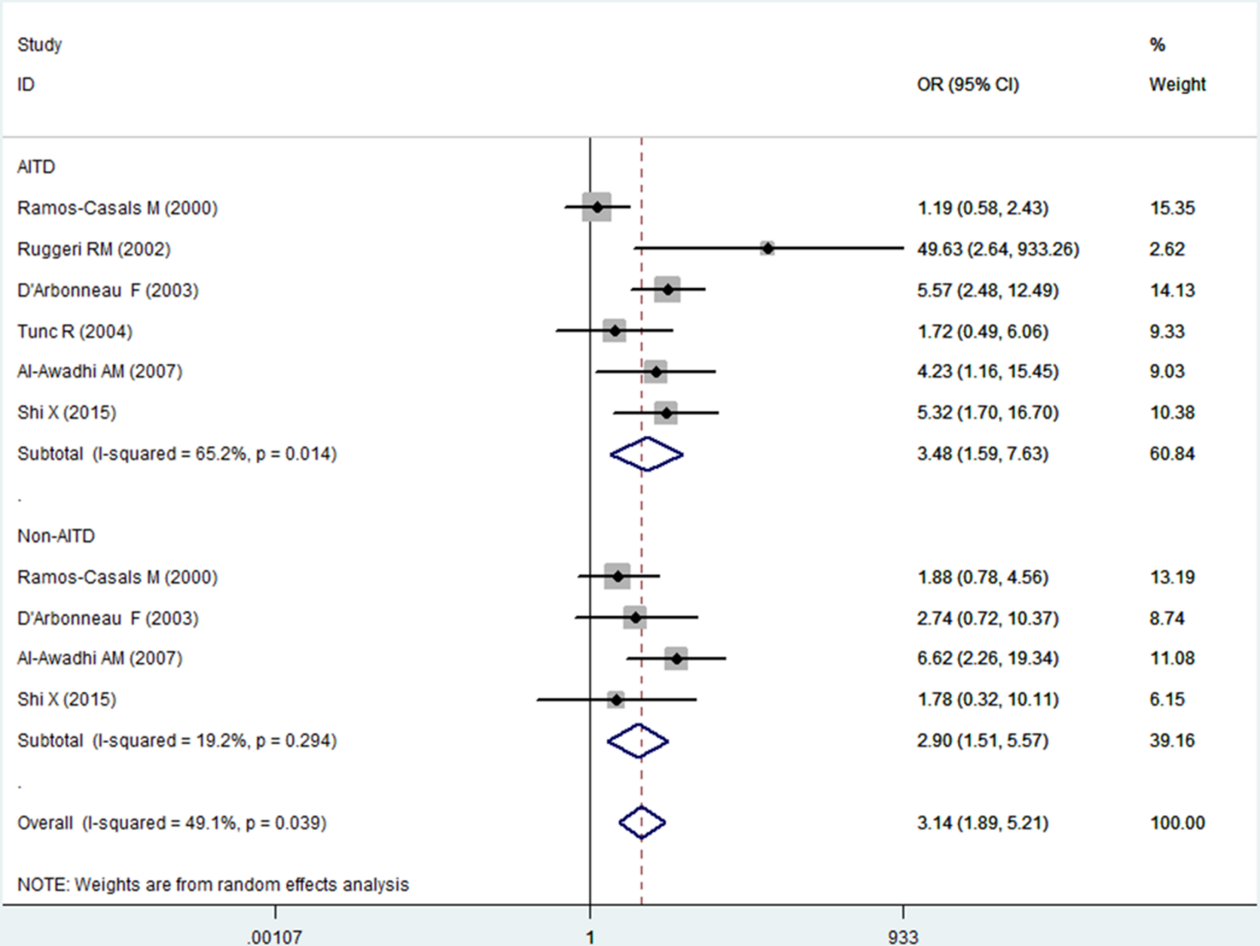
Forest plots of the frequency of autoimmune thyroid disease (AITD) and non-AITD in patients with Sjogren’s syndrome compared with the controls. Diamond, pooled odds ratio (OR), and 95% confidence interval (CI).

**Figure 4:**
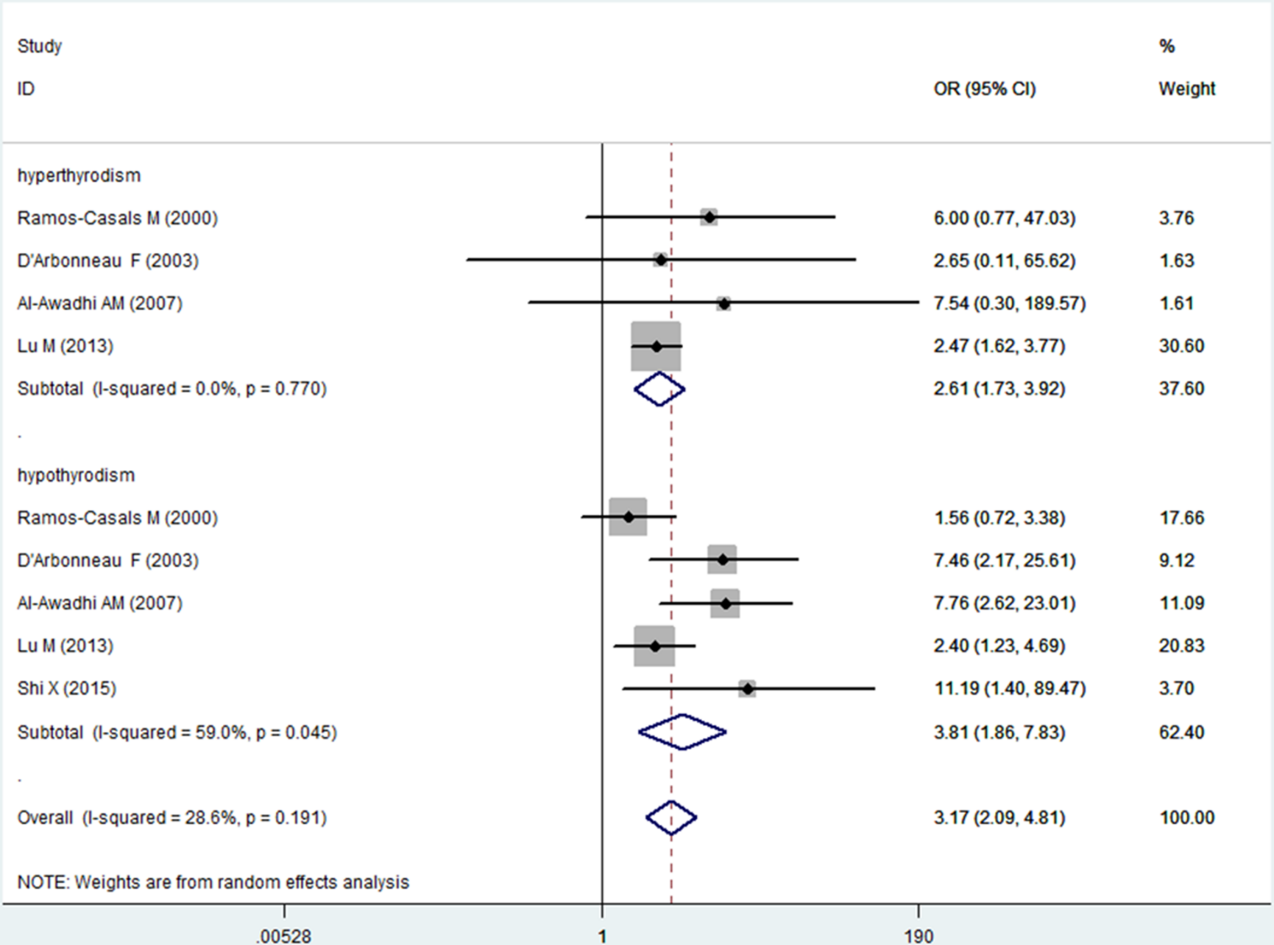
Forest plots of the frequency of hyperthyroidism and hypothyroidism in patients with Sjogren’s syndrome versus controls. Diamond, pooled odds ratio (OR), and 95% confidence interval (CI).

The risk of AITD was higher in patients with SS than in controls (OR, 3.48; 95% CI [1.59–7.63]), and the risk of non-AITD was also higher in patients with SS than in controls (OR, 2.90; 95% CI [1.51–5.57]) (Fig. 3). Furthermore, we investigated the relationship between abnormal thyroid function and SS. The risks of both hyperthyroidism and hypothyroidism were higher in patients with SS than in controls (hyperthyroidism: OR, 2.61; 95% CI [1.73–3.92]; *I*^2^ = 0%; hypothyroidism: OR, 3.81; 95% CI [1.86–7.83]; *I*^2^ = 59.0%) (Fig. 4).

### Sensitivity analysis and publication bias

**Figure 5:**
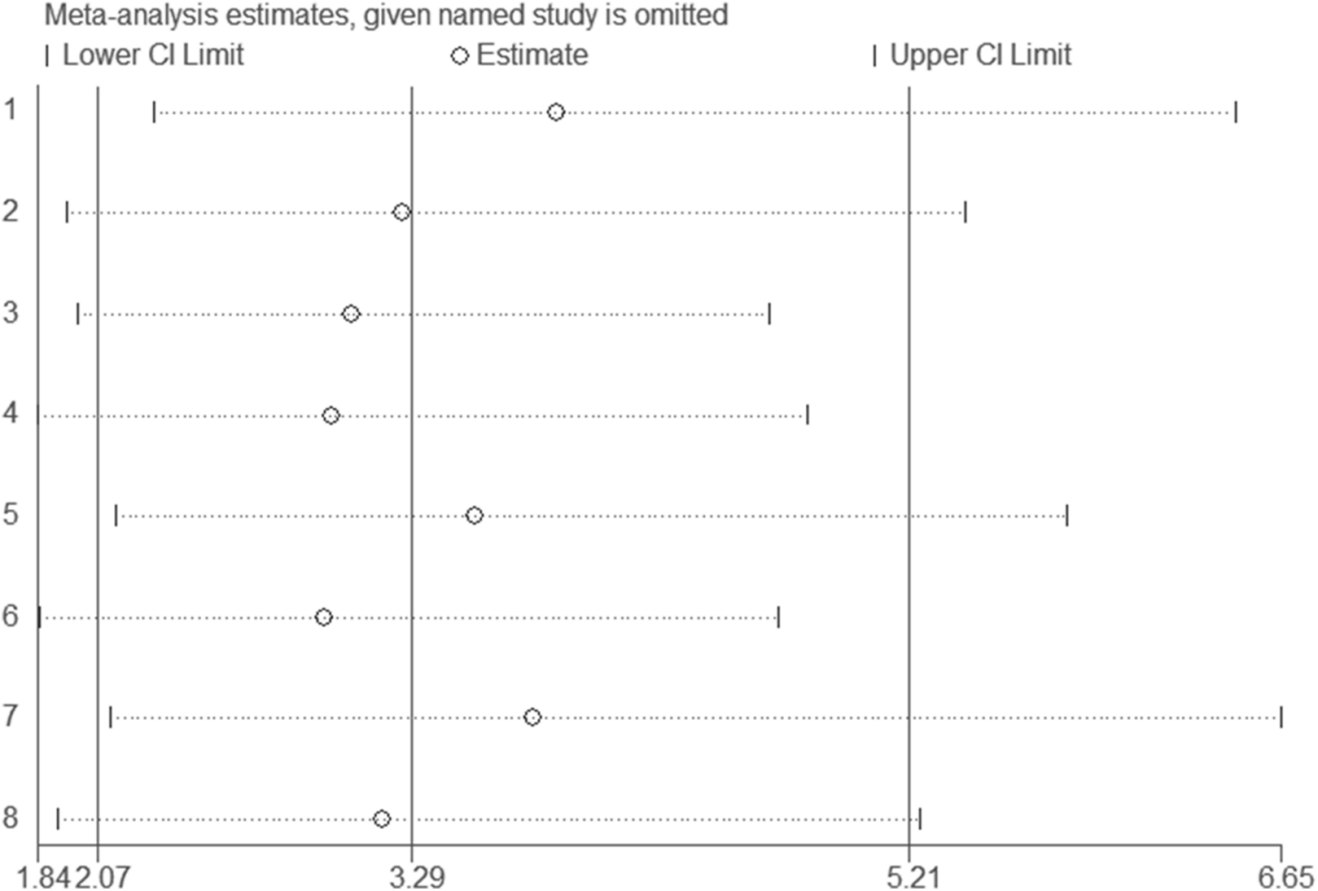
Sensitivity analysis results. The sensitivity analysis was performed to examine the influence of any particular study. There was no significant difference in results of the sensitivity analysis and our previous estimates, indicating that our statistics were relatively credible.

**Figure 6:**
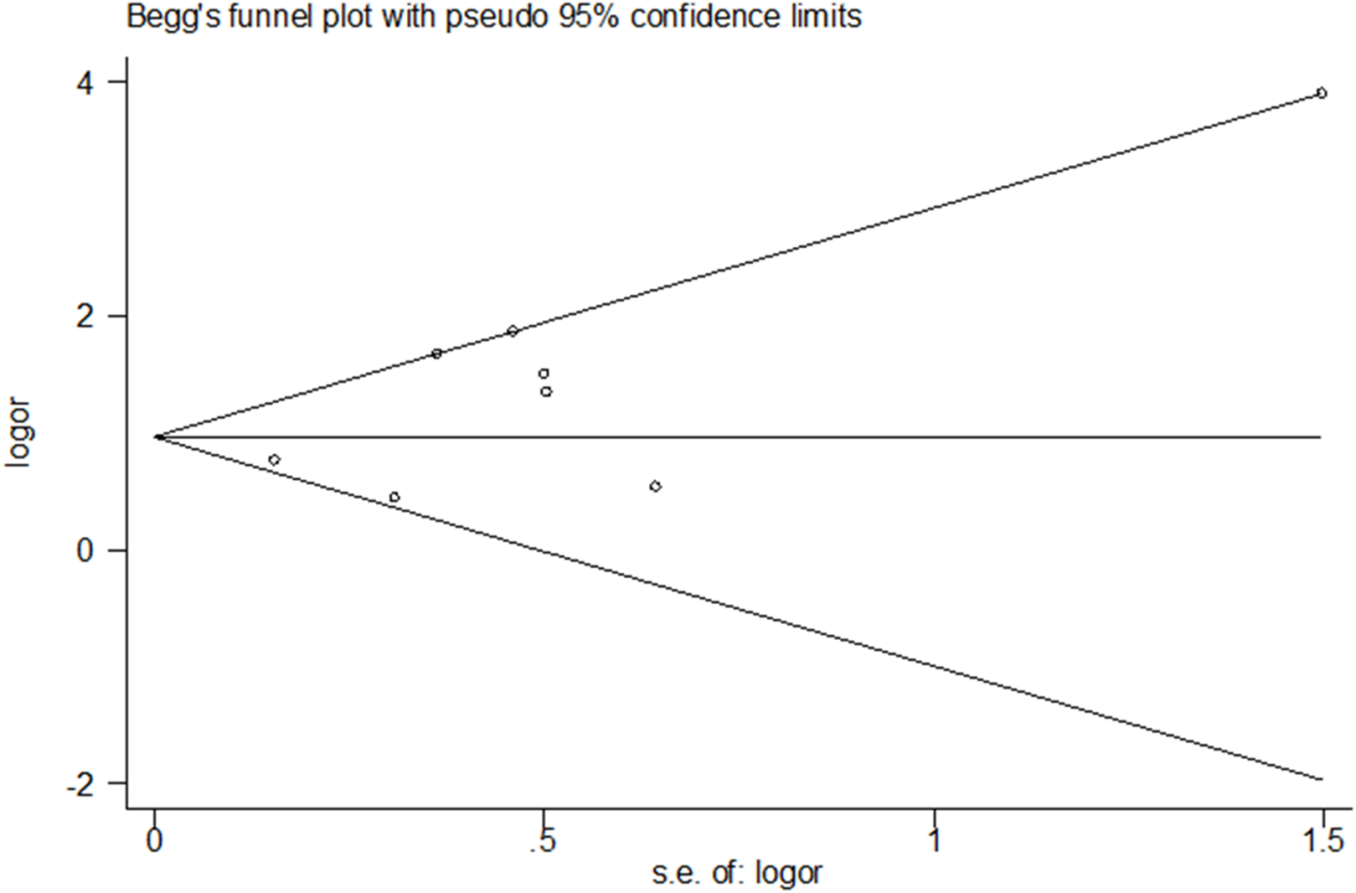
Publication bias results. Begg’s test was used to determine whether potential publication bias existed in the reviewed studies. The results suggested that there were no publication bias.

A sensitivity analysis was performed to examine the influence of any particular study (Fig. 5). There was no significant difference in results of the sensitivity analysis and our previous estimates, indicating that our statistics are relatively credible. We carefully and comprehensively searched the articles obtained from the databases and obtained research details from the authors. We also performed Begg’s test to determine whether potential publication bias existed in the reviewed studies. The results (*P* = 0.71) suggested a lack of publication bias (Fig. 6).

## Discussion

To our knowledge, this systematic review is the first to estimate the risk of thyroid disease in SS patients. Some studies assessed the risk of thyroid disease in SS patients, but the results of these studies were inconsistent. Because of the relatively small sample sizes of these studies, it was difficult to determine the relationship between SS and thyroid disease. This review summarizes and quantitatively evaluates the evidence of an increased risk of thyroid disease in SS patients. A total of eight independent studies were included in this meta-analysis. The results showed that the incidence of thyroid disease was significantly higher in SS patients than in controls (OR, 3.29; 95% CI [2.08–5.21]).

From a genetic point of view, the expressions of HLA-DR3 (HLA-DRB1*03:01), HLA-DQ (HLA-DQA1*05:01, HLA-DQB1*02:01), and HLA-B8 are higher in AITD (Effraimidis & Wiersinga, 2014). They are susceptible genes for SS, while HLA-DR3 in particular is associated with positive anti-SS-B and anti-Ro-52 antibodies (Cruz-Tapias et al., 2012). This indicates that SS and AITD share the same pathogenic basis at the gene level. However, although genetic factors play an important role in the pathogenesis of both diseases, the aforementioned susceptibility genes are also expressed in healthy people, which indicates the role of non-genetic factors in their pathogenesis. Age, sex, and hormone levels were identified as endogenous predisposing factors for SS and AITD (Amador-Patarroyo et al., 2012). Some studies showed that the prevalence of SS and AITD increased with age. Age-induced increases in the incidence of disease may be due to aging-induced damage to the innate and acquired immune system, resulting in a decline in the body’s ability to respond to antigen stimuli as well as changes in the number and activity of immune cells and cytokine levels. SS and AITD tend to affect female patients, particularly perimenopausal women, which indicates the effect of hormones on SS and AITD (Anaya et al., 2016, 2018).

However, the prevalence of non-AITD is also high in patients with SS. This indicates that the immunological mechanism is not the only reason for the high incidence of thyroid diseases in SS patients. Thyroid, salivary, and lachrymal glands are quite similar from a histological and functional point of view (Mason, Harden & Alexander, 1967). Smoking, viruses, and environmental factors are risk factors for the two diseases, while the mechanism of their occurrence requires further research.

The main purpose of this meta-analysis was to statistically investigate the risk of thyroid disease in SS patients. However, our meta-analysis has some limitations. Due to the lack of randomized controlled trials, only case-control or retrospective studies were included in this meta-analysis. In addition, although women are more likely to suffer from thyroid disease, the studies included in this meta-analysis had different sex ratios, which might have influenced the estimation of the overall risk of thyroid disease in patients with SS. Some studies used patients with other diseases such as osteoarthrosis as controls instead of healthy volunteers, which also might have influenced the results.

## Conclusion

To the best of our knowledge, this systematic review is the first to estimate the risk of thyroid disease in SS patients. The results of our meta-analysis support the hypothesis that the risk of thyroid disease is increased in patients with SS than in controls, which suggests that SS patients should be screened for thyroid disease.

## Supplemental Information

10.7717/peerj.6737/supp-1 Supplemental Information 1 PRISMA checklist.

10.7717/peerj.6737/supp-2 Supplemental Information 2 Rationale and contribution of meta-analysis.

## Additional Information and Declarations

### Data Availability

The following information was supplied regarding data availability: The raw data are available in Tables 1 and 2.
