# Supplementary material for: Increased risk of thyroid disease in patients with Sjogren's syndrome: a systematic review and meta-analysis
Source: PeerJ. 2019 Mar 19;7:e6737. doi: 10.7717/peerj.6737 (PMC6430100; doi:10.7717/peerj.6737)
Supplement: Supplemental Information 2 [file peerj-07-6737-s002.docx]

1. **The rationale for conducting the meta-analysis**

Sjogren's syndrome (SS) is an autoimmune disease involving the exocrine glands. The typical clinical manifestations are persistent dry mouth and eyes. And some female patients may even have vaginal dryness. The disease can lead to multiple systems and organs damage, especially in the lungs, kidneys, and blood system. The initial study suggested that SS with thyroid disease is not common, but as the incidence of SS continues to rise, more and more Sjogren's syndrome with thyroid disease appears. The risk of AITD in SS patients may be higher than the controls. However, there is lack of agreement on the prevalence of thyroid disease and non-AITD in SS patients.

1. **The contribution that the meta-analysis makes to knowledge in light of previously published related reports, including other meta-analyses and systematic reviews**

To the best of our knowledge, this systematic review is the first to estimate the risk of thyroid disease in SS patients. The results of our meta-analysis support the hypothesis that the risk of thyroid disease is increased in patients with SS compared with the controls, which suggests that SS patients should be screened for thyroid disease.
